# GP/GPN partner* perspectives on clinical placements for student nurses in general practice: can a community of practice help to change the prevailing culture within general practice?

**DOI:** 10.1186/s12875-018-0842-2

**Published:** 2018-09-08

**Authors:** Robin Lewis, Shona Kelly

**Affiliations:** 1 Department of Nursing and Midwifery, Sheffield Hallam University, Sheffield, S10 2BP England; 2 Department of Social Work, Social Care and Community Services, Sheffield Hallam University, Collegiate Crescent, Sheffield, S10 2BP England

**Keywords:** General practice, Workforce, Placements, Nurse education, Organisational culture, Community of practice

## Abstract

**Background:**

The UK Government document *5 year forward view* describes the need to move chronic disease management from secondary to primary care, which will require a significant increase in the numbers of General Practice Nurses (GPNs). Until recently, there has been no specific recruitment strategy to address this increased need. In recent times, a number of solutions have been suggested to address this impending GPN recruitment crisis. For example, Health Education England (HEE) commission General Practitioners (GPs), who are members of the Advanced Training Practice Scheme (ATPS), to provide placements for student nurses within general practice.

**Methods:**

A descriptive qualitative study was undertaken, in which data were collected using semi-structured interviews with 16 GPs and 2 GPN partners*. Qualitative analysis used a framework approach and themes were cross-checked within the team and member checking was undertaken with a convenience sample of GPs. The research had ethical approval and anonymity and confidentiality were maintained.

**Results:**

From the GP perspective, there were two key themes that emerged from the data. The first theme of ‘*fishing in the same small pond’* included succession planning for the general practice workforce, the ‘merry go round’ of poaching staff from other practices, and the myths and misunderstandings that have grown up around general practice nursing. The second theme, ‘*growing your own’,* looked at the impact of the student nurse placements as a means to address the crisis in GPN recruitment. There was recognition of the need for cultural change in the way that GPNs are recruited, and that the ATPS was one way of helping to achieve that change. There were however a number of challenges to sustaining this cultural shift, such as the financial constrains placed upon the GP practice, and the need to function as a ‘small business’.

**Conclusions:**

Despite all the challenges, the evidence is that, through the Community of Practice (CoP), the ATPS scheme is beginning to ‘bear fruit’, and there is a subtle but discernible move by GPs from a ‘why *would* we?’ to ‘why *wouldn’t* we?’ invest in education and training for nurses in general practice.

N.B. The term GPN partner* denotes a GPN who is a ‘full partner’ in the practice business, holding the same NHS contracts and the same status as a GP. For the purposes of the paper itself, the term GP will be used to denote both types of partner.

**Electronic supplementary material:**

The online version of this article (10.1186/s12875-018-0842-2) contains supplementary material, which is available to authorized users.

## Background

There is constant reference in the UK media to an ‘impending workforce crisis’ in UK general practice, set against the backdrop of the Health Education England (HEE) report into primary care [1] and the Royal College of General Practitioners (RCGP) Practice Forum Report [2]. Both of these reports concluded that General Practitioner (GP) partners are finding it harder to recruit GP trainees and to replace those GPs who are increasingly opting to retire. This occurs at the same time as the current ‘direction of travel’ within NHS England is to move more and more services from secondary care into primary care; in particular chronic disease management. NHS England’s *5 Year Forward View* [3] outlined plans to move more services into the community however it is acknowledged that if general practice is to be able to meet this future demand, the necessary human resources must be put in place to support that transition.

General practitioners (GPs) are doctors that work in primary care. They treat common medical conditions and refer patients to hospital and other services for urgent and specialist treatment. In some parts of the UK there is a significant shortfall in the number of General Practitioners (GPs), and the age profiles of GPs are such that there is significant concern over the supply of appropriately trained GPs to fill future vacancies. Despite initiatives to increase the numbers of GP training opportunities available, applications to GP training nationally continue to fall. Some parts of the UK particularly in the north of England, significantly struggle to recruit sufficient numbers of GP trainees [4]. This chronic shortage of GPs has had a number of consequences for general practice, specifically the enhanced profile of the General Practice Nurse (GPN) [5]. Central to this study, the GPN role has evolved and developed to address some of these workforce issues. In particular, chronic disease management and surveillance now comes under the aegis of the GPN [7].

This has resulted in GPNs taking on more responsibility for running long term conditions clinics such as those for chronic asthma and diabetes [6, 7]. This ‘extended’ clinical responsibility requires specialist education and training, including training to ‘independently prescribe’ medications [6, 7]. However, the age profile of GPNs is not dissimilar to those of GPs, with a recent Queen’s Nursing Institute (QNI) report [8] identifying that approximately 33% of GPNs are due to retire within the next 5 years. If there is no clear recruitment and retention strategy in place to increase the numbers of GPNs to both replace those GPNs due to retire and to address the increasing workload being transferred to general practice, then there is a ‘perfect storm’ brewing in which there will be an acute shortage of both GPs and GPNs at a time when the workload in primary care will be at its greatest [5].

It has been argued [9–13] that at least part of the forthcoming GPN recruitment crisis relates to the dearth of general practice placements available for student nurses in the UK. Unlike medicine, there is no established culture of student nurses spending time on placement in general practice. This means that many student nurses do not know what general practice nursing is or what it has to offer, and conversely many GPs are unaware of the current situation in nurse education [9]. This clearly needs to be addressed, and one practical way to do this is to increase the number of clinical placements for student nurses within general practice. However, the QNI survey [8] also found that only 27% of GPs offered placements for undergraduate nursing students, compared to 61.5% offering placements to undergraduate medical students.

It is clear that these matters are not unique to the UK. The Australian government has similarly invested in providing incentives for general practices to employ more GPNs, however the same issues remain as in the UK [14]. The move to treat an ageing population with increasingly complex healthcare needs in primary care, allied to challenges in addressing shortages in the primary care workforce mean that Australia is facing many of the issues being addressed here. In New Zealand, the picture is slightly different. The development of a Primary Health Care Strategy [15] in 2001 highlighted the need to invest in the development of Practice Nursing as a career, with an associated national career pathway for GPNs. This means that in New Zealand, significant advances have been made in recent times to both recruit and retain GPNs.

In recent times, there have been a number of proposed solutions to the shortage of student nurse placements in general practice, and to positively influence the developing GPN recruitment crisis in the UK [9–13]. In a number of areas within the UK, GPs have been specifically commissioned by HEE to provide placements for student nurses. These schemes are part of the wider National Training Hubs Initiative (NTHI). This is a targeted response to two key government documents: The *GP Forward View* [16] and The *5 Year Forward View* [3]. It brings together primary care and higher education institutions such as Sheffield Hallam University in the UK, enabling the development of the networks required to support future workforce planning. As part of the NTHI, this Advanced Training Practice Scheme (ATPS) within the north of the UK provides General Practice (GP) placements for undergraduate student nurses that offer opportunities for students to gain experience in a general practice setting. In addition, further workforce transformation initiatives such as the ‘GPN Ready’ scheme (to support the recruitment of new graduate nurses) have also been developed under the ATPS banner. This particular ATPS covers a large geographical area in the North of the UK, which is predominantly urban in nature, with a population with higher than average indices of deprivation [15]. It is a partnership between the participating GP practices and SHU, and is specifically designed to provide opportunities for student nurses at all levels of training to gain an in-depth and sustained exposure to, and experience of, general practice nursing [13]. The idea is that this dedicated exposure to general practice will encourage the students to consider applying for a GPN post on graduation.

Students’ career intentions upon graduation are influenced by their perceptions of the different clinical areas in which they work [17]. Popular clinical environments such as those in acute care are viewed as being dynamic, exciting and challenging places in which to work, and much of this information appears to come from students’ working knowledge of the area and from positive placement experiences. Consequently, there has been no real opportunity for students to positively experience general practice, and all the evidence points to a lack of knowledge and understanding of the role of the GPN and all that it has to offer.

This is borne out by the findings from a study carried out by Bloomfield et al. [18] which looked at students’ career intentions in relation to primary care in Australia. The study compared tertiary care, secondary care and primary care as potential career options. The authors found that primary care was not regarded seriously as a viable career option by the majority of the students that were surveyed. In addition, there have been a number of studies from Australia that have looked specifically at students’ experiences of general practice placements [19, 20]. The findings from these studies reported a generally positive student experience, and although the placements did provide some impetus for consideration of a career in general practice, little is currently known regarding the true impact of primary care placements upon students’ career intentions or on the GPs themselves.

### Communities of practice (CoP)

The theoretical approach used in this study is based upon Lave & Wenger’s idea of a ‘community of practice’ (CoP) [21]. According to Lave & Wenger, a community of practice (CoP) is a *socially situated, practice-based* approach to learning, in which the learning that takes place is viewed in terms of a series of collective, relational and social processes (as opposed to an individual, solitary process of knowledge acquisition). It involves a complex relationship between novice(s) and expert(s), being socialised into the practice and developing an identity within that practice community.

As a theoretical lens through which to view the general practice placements, CoP are clearly attractive because they use a *socially constructed* approach to learning, in that learning is seen as much more than simply the individual acquisition of knowledge [21]. It is argued that the CoP as a community of learning enables the development of positive personal relationships and ways of interacting, together with a mutually constructed sense of identity. As Ranmuthugala et al. note [22], a strong learning community promotes healthy interactions and work-based relationships based upon mutual trust and respect. The successful CoP may be characterised in terms of a shared *domain* of interest, a *community* that pursues that shared interest and the open and unbiased *sharing* of knowledge, information and resources. The development of a work-based identity is key, in that it enables the GPs to visualise how the neophyte GPN might ‘fit in’ and positively influence the general practice team [23].

By adopting a socially constructed approach to learning, learning is seen as much more than simply the acquisition of knowledge. Therefore, by using the CoP we may gain a better understanding of the processes by which the GPs’ views and perceptions are formed. This will enable us to address the key aims of the study, which are outlined below:

### Aims and objectives

The overarching aim of the study was to explore GP perceptions of the student nurse placement in general practice. There were a number of specific objectives relating to the examination of:

1. GPN recruitment: culture and practice
2. The effectiveness of the ATPS ‘model’ of student nurse placements
3. General Practice as a career for new graduate nurses

## Methods

### Context for the study

The ATPS being studied was one of the first of the Practice placement networks to be formed, and has been in existence since 2009 [1]. The overarching philosophy of the scheme is to promote sustainable cultural change in general practice, by widening student nurse access to general practice placements. Through the ATPS, GPs are also supported to ‘grown their own’ GPNs by recruiting new graduate nurses. As the ATPS becomes fully embedded, the idea of ‘growing your own’ is beginning to change the prevailing culture within general practice. Partner HEIs such as SHU work closely with the GPs to provide support for the students (proto-GPNs) through the ATPS placements and the recruitment of new graduates into a GPN post (neophyte GPN) and afterwards (emergent GPN).

### Study design

A descriptive, qualitative approach was used for the study. This involved the use of semi-structured, one to one interviews with 16 GPs and 2 GPN partners. Although it may be seen as a ‘low inference’ approach to interpretation of the data, the descriptive qualitative study design is a pragmatic way for the researcher to gain an authentic and in-depth insight into the perceptions, thoughts and experiences of the participants. This type of study design may be regarded as a useful means for addressing many research questions in health care, since it is an effective way in which to look at the experiences of health professionals and their views on the way that health care systems are organised.

### Cohort and sample

A purposive, cross-sectional sample of GPs was drawn from the population of 37 practices currently participating in the ATPS programme. The GPs in these practices were approached by letter and a follow up phone call. The sample was based upon data relating to the number of GPs and GPNs within the practice and the length of time the practice has participated in the ATPS programme.

### Data collection

Data collection was carried out using semi-structured interviews with an interview schedule (please refer to Additional file 1 for details) based upon the findings from a rapid review of the existing literature. There were seven questions in the interview schedule, and these related to the views of the GP on general practice as a placement for student nurses. The questions were used as prompts for the interviewer and as a framework to guide the dialogue. The interviews were conducted by a member of the study team and took place at a date and time of the participants’ choosing. With the participant’s consent the interviews were digitally recorded, with each interview lasting approximately 15–20 min. Data collection continued until saturation was reached.

### Research governance

Following an agreement in principle to take part, formal written consent was obtained from all interview participants prior to the interviews taking place. Ethical approval for the study was obtained from the SHU Faculty Research Ethics Committee (Ref: H447). SHU Research governance protocols were adhered to throughout the study. All data was anonymised to maintain confidentiality and to ensure that no individual could be recognised in any subsequent report. Paper based data is kept securely in a locked drawer and electronic data and information relating to this research is kept on a password-protected computer on a network storage system that adheres to Home Office Standards of Data Security. This data will be kept for a minimum of 7 years in accordance with SHU guidelines.

### Data analysis

The interview transcripts were anonymised and each transcript was allocated a unique code. They were entered into Quirkos© software for analysis and cross-checked for accuracy by the team. Once it had been cross-checked, the data was analysed following the National Centre for Social Research ‘Framework’ guidelines [24]. This approach has emerged from applied health and social policy research and analysis. It involves a systematic processing, sifting, charting and sorting of material into key issues and themes. It also permits both within- and across-case comparisons and allows the integration of existing knowledge from previous research and policy into the emerging analysis. All transcripts were analysed independently by all four members of the research team and the interpretation of data was also cross-checked within the team. Member checking was also used, as were direct quotes from the participants. The use of these techniques both enriches and enhances the link between the respondents’ experiences and the analysis of the data (25).

## Results

There were two key themes that emerged from the data. The first theme (Table 1) looked back at what had gone before, with the recognition that things needed to change. This included issues such as the culture of general practice, the need for succession planning and the poaching of experienced staff from other practices. The second theme (Table 2) looked at the impact of the student nurse placements. This included the need to address the longstanding cultural issues and the need to invest in the GPN workforce.

**Table 1:** Themes and sub-themes

Theme One:	Sub-themes:
*“Fishing in the same small pond”*	• Succession planning
	• The recruitment ‘merry go round’
	• Cultural antecedents
	• Adapt to survive

**Table 2:** Themes and sub-themes

Theme Two:	Sub-themes:
*“Growing your own staff”*	• The need for ‘new blood’
	• The need for some fine tuning
	• Investing in the future workforce
	• Green shoots of cultural change

### Fishing in the same small pond

The first theme of ‘*fishing in the same small pond’* included the lack of infrastructure and succession planning for the general practice workforce, the ‘merry go round’ of poaching staff from other practices and the various misunderstandings that have grown up around general practice nursing (see Table 1). The care delivery ‘model’ for general practice has remained largely unchanged since the inception of the NHS and general practice continues to be run by small, independently-run businesses, contracted to provide a portfolio of defined health services to a registered list of patients. These businesses are owned and run by one or more GP/GPN ‘partners’ who hold the various NHS patient care contract(s).

#### Succession planning

For the most part the culture and practice of GPN recruitment had also not fundamentally changed in decades. The age profile of both GPs and GPNs within the UK meant that a significant proportion of the GP and GPN population were nearing retirement age. From a practical point of view, this GP commented on the ‘perfect storm’ that was brewing within general practice:

*"… there are lots of GPs round here who are coming up to retirement, and they have all got practice nurses that are coming up to retirement too …"*

This GP was also pragmatic in the approach to GPN recruitment. The difficulties in recruitment were clear, and the GP acknowledged the need for a fresh look at the subject:

*"We had some real difficulty filling that last vacancy … we've got a number of people* [GPNs] *approaching retirement and that gives us an opportunity to perhaps do things a bit differently next time …"*

When a GPN leaves a practice there is a hiatus caused by the recruitment processes. The economies of scale in general practice (i.e. small to medium businesses working in small, socially isolated teams) mean that when they do recruit a GPN, the practice will attempt to recruit an experienced, competent nurse who won’t need much training and can therefore ‘hit the ground running’. Inevitably this desire simply reinforces the idea that newly qualified nurses and general practice are not suited to each other. Older, more experienced nurses were often recruited from secondary care settings. There were however, a number of reservations voiced regarding the motives of these nurses. For example, this GP commented:

*"I think* [the] *hospital nurses want to come into primary care because it's a Monday to Friday nine-to-five job … they've done a few years in hospital with shifts and nights and everything else, seven days a week and then they start thinking, actually, I quite fancy a job that's a bit more family-friendly. It's not always* … *'I really want to work in primary care'…"*

#### The recruitment ‘merry go round’

In spite of the numbers of nurses recruited from secondary care, experienced GPNs remained at a premium. The dwindling numbers of GPNs and the increasing need for skilled, experienced nurses to take on chronic disease management to meet UK Government monitoring (QOF: Quality Outcomes Framework) targets meant that they were (and still are) much in demand. This situation has led to a GPN recruitment ‘merry go round’. In an assessment of the current situation, this GP remarked:

*"There's going to be a much greater demand for practice nurses and particularly the more highly skilled ones that can manage their own caseload …"*

It is not difficult to see how and why the ‘merry go round’ has existed to date, but there was a realisation that it was becoming unsustainable:

*"I do understand that we are poaching them* [GPNs] *from each other … You know, obviously, there’s not enough nurses around* (sic) *and it does seem to me they just swap from one practice to another … "*

This GP also reflected upon the futile nature of ‘poaching’ of GPNs from other practices. There was also recognition of the increasing difficulties in recruiting experienced GPNs.

*"They just poach from other practices, but* [even] *that’s dried up now … they’ve been taking from secondary care too … So we're all just fishing in the same small pond, aren’t we?"*

#### Cultural antecedents

The current situation clearly has a number of historical and cultural antecedents. There has been an historic lack of communication between general practice and nurse education, and this has led to a number of misunderstandings. For example, this GP expressed frustration over the current situation:

*"We've had to work hard to get an understanding from some of the GPs and from quite a lot of university teachers and from quite a lot of student nurses too that they* [nurses] do not* *need secondary care experience before they go into primary care ... and that’s the problem"*

* Denotes heavy emphasis placed on these words by respondent

There was also a widespread frustration amongst the GPs that most of the nursing students’ placement time was still spent on hospital wards. Although the balance between the number of primary and secondary placements is slowly changing, the comment of this GP was typical:

*"I don’t know how many of the students will actually try and get into general practice… most of them go into the hospitals* [when they qualify] *as that’s where they have all their training…"*

Working in acute care was also perceived as being much more much more attractive to the students than working in primary care. This GP noted ruefully:

*" … They all seem to think that working in a hospital is sexier* (sic) *than working in a GP surgery …"*

This then became a self-fulfilling prophesy in which GPs did not actively seek to recruit new graduate nurses, and new graduate nurses did not apply for GPN posts. However, the findings from another component of this evaluation [20] indicated that the students with general practice placements were beginning to appreciate the role of the GPN and the opportunities that it provided. The prospect of working in general practice upon graduation was now seen as attractive to many of them.

#### Adapt to survive

There did seem to be an acknowledgement of the need to change the culture of GPN recruitment and retention. The demands for new ways of working proposed in the *5 Year Forward View*, together with the longstanding difficulties in GP recruitment meant that the skill mix within general practice that had existed for years needed to be updated to meet the requirements of the twenty-first century patient. The GPs were candid in their assessment of the need to ‘adapt to survive’:

*"I mean … 'traditional' general practice needs to adapt … previously you had say five or six GPs, a couple of nurses and one HCA* [in a practice] *but the skill mix is not needed like that anymore … "*

This GP went on to say that:

*"… there are some practices who are already beginning to change, so they'll have three GPs, four or five nurses, of which three will prescribe and one's an ACP* [Advanced Clinical Practitioner], *and five or six HCAs"*

The development of non-medical staff within general practice has been ad hoc and informal, and this has meant that the infrastructure required for sustainable workforce change has not been a priority. The accelerated expansion of non-medical roles in more recent times has resulted in a ‘patchwork’ of different roles and different titles. The introduction of the ACP role is a good example of this. The title of ACP is not regulated in the same way as the title of GP, but an ACP is generally considered to be a fully autonomous practitioner, educated to Masters Level, managing their own caseload in the same way as the GP.

However a number of experienced GPNs had already developed a high level of autonomy through for example the ability to independently prescribe medication. There was however the realisation that this cohort of GPNs was dwindling due to retirement and that the traditional recruitment methods were becoming obsolete. Although experienced GPNs were highly sought after, they were seen to have their drawbacks. This GPN partner commented:

*"What you don't want is loads of other people's 'baggage', which you generally get with somebody who's come from somewhere else … 'we don't do it that way; I do it this way'… far better then, to recruit newly qualifieds* (sic) *and 'grow your own'?"*

### Growing your own staff

The second theme ‘*growing your own’,* looked at the impact of the student nurse placements upon the looming workforce crisis. As we can see from the first theme, there was already acknowledgement of the need for cultural change, and in this second theme, recognition that the ATPS was one way to achieve that change. There were however a number of challenges to this, including the often competing demands of the GPNs for education and the practice as a ‘small business’.

The findings here (see Table 2) show that the philosophical approach of ‘growing your own’ GPNs had begun to gain some traction, albeit on a small scale, within general practice. This GP in particular could see the benefits of ‘growing your own’ GPNs, and was also clear that the current situation was becoming untenable. There was a need for ‘new blood’.

*" … GPs need to understand that if they want a new nurse, they can either try and poach one off* [the] *'roundabout', which just recycles what's there, does nothing for the gene pool or they can take somebody straight from training* … *and train them up themselves …"*

Through the CoP, the student nurses were beginning to have a small but discernible impact on general practice. One GP summarised the benefits of the ATPS scheme. From a partner’s point of view, the new graduate nurses would be free of any ‘bad habits’, unlike some of the GPNs they had previously employed.

*"I suppose they’ll not come with any of the preconceived ideas of, you know, 'this happens in the practice that I used to work in before'… from the beginning you’re teaching them how you want it to work here from the very start. I think that’s got to be a good thing…"*

Again, there was recognition that ‘new blood’ was needed. This GP was clear that there was a need to reinvigorate the GPN role, and to attract new, dynamic graduate nurses:

*"We’ve got to encourage the younger generation… I think we’ve got this fixed idea of what practice nurses are, and we think of the 'old school' nurses and so many things have changed now… So I think we would encourage new young nurses to come in…"*

There were a number of other benefits highlighted by the respondents. For example, the influence of the CoP was such that the student nurses kept the other staff ‘on their toes’. As this GP astutely noted:

*"I think there are several advantages* [to the ATPS] *having somebody helps you keep up to date* […] *that also helps the practice* […] *It gives a different perspective because the nurses that are training are doing other things* […] *so they’ve got a slightly different angle on things..."*

There was also recognition of the need for change, and the benefits that younger, newly graduated staff would bring to the practices. Commenting upon the presence of the student nurses in her practice, this GP was enthusiastic about the possibilities:

*"I think they* [the student nurses] *bring a fresh and different outlook, different skills too… I would hope that we’d be able to develop them and they'd stay… like I say it’s growing your own isn't it..?"*

This quotation seemed to encapsulate the developing influence of the ATPS and the new CoP. Finally, the parallels with medical training placements were becoming clear. As this GP noted:

*It's a win-win situation* [the ATPS]*. If they like the practice and you like them, then you know they're coming up… that's what we do with the* [GP] *trainees. I mean most of the partners here have been ex-trainees in this practice because it's much better to recruit someone you know who's worked here. And they know what you're like so it's about getting the right fit, isn’t it?"*

#### The need for some ‘fine tuning’

Although the CoP generated by the ATPS placements was generally seen as ‘a good thing’ by the majority of the respondents, there were still a number of issues to be addressed. Initially, the students undertook 6 week placements which were deemed to be the minimum length of time for the students to gain sufficient exposure to general practice to make an informed decision about the placement. This GP argued for longer placements to be the norm:

*"We are trying to address it* [the shortage of GPNs]*. We're taking student nurses now and that's great. But they only came for like a six week block in a three-year training programme. It's not enough. We need them for longer but I don’t know how we would get around that* […] *I think they do need more exposure to primary care…"*

Despite the progress being made through the CoP, there was still some evidence of a residual lack of understanding between general practice and nurse education. Having experienced the ATPS, a number of the GPs wanted the students to return each year for a placement. This GP was clear what was needed for the practice to grow its own GPNs.

*"Realistically, if you really wanted grow your own, you would have them in the first year, then the second year and then you would have them back in the third year, back to you at your practice, and then you'd offer them a job at the end of it. That’s where this* [ATPS] *would come in…"*

This GP was clear that the partnership between general practice and the HEIs providing nurse education are vital to the long term sustainability of the ATPS. The GP identified that there was still some ‘room for improvement’.

*"It needs to be a proper partnership between you* [the HEIs] *and us then we all benefit don't we? I don't think we understand enough* [about each other] *but having them* [the students] *helps…"*

#### Investing in the future workforce

In the UK, the education and training for GPNs is funded primarily by the business. There is some limited access to government funding; however this tends to be regional and ad hoc. The issues over the funding of education meant that there were still some challenges to the recruitment of new graduate nurses. For example, this GP was worried that a newly qualified nurse would not stay in post. Having been used to longevity in their practice staff (one of the GPNs had been with their practice for over 20 years), this was clearly a concern.

*"… I think there’s perhaps a worry that somebody at that* [early] *stage of their career is more likely to be looking for the next job…"*

Another concern was the disparity between what hours the newly qualified nurses would expect and what hours would be available. Given that the GPN role has historically not been the domain of newly-qualified RNs, this is not surprising. Stereotypically, older nurses with children will generally prefer part time, child-friendly working patterns. This very subtly feeds into the idea that general practice is no place for young, new graduate nurses. As this GP noted:

*"The younger ones… they'll all want full time hours… you are going to get* [some] *smaller practices who don't always need full-time nurses… then what?"*

From a strategic workforce perspective, the ATPS placements may be seen as the first phase of a much wider, long term project to address the recruitment and retention of GPNs in general practice. Once recruited, the need for a more formalised framework of education and training for new graduate GPNs was also becoming clear, albeit rather slowly. Inevitably, this raised the issue of the financial investment required for nurse education and training. The fact that general practices are medically-dominated small businesses means that the provision of education for non-medical staff has not always been a high priority and the needs of the practice were always seen as taking precedence. As this GP noted when asked about financial support for neophyte GPN education and training:

*"We'd have to think… 'what would that enable them to do and does it fit with what we want them to do?'…"*

There was a clear tension between the needs of the practice and the needs of the staff. This was made clear by one of the GPs who said:

*"We're a business in the same way everyone else is* (sic) *and therefore it's not like a hospital*… *you're not going to train someone at your own cost are you?"*

This was a recurrent theme, linked to the GPN recruitment ‘merry go round’ already described. Clearly this had happened before. Although expressed in a rather convoluted manner, this GP did make the point that:

*"… it would be a disincentive to encourage increasing training* (sic) *if you think that somebody who’s better trained might get poached by another practice in order to get a pay rise…"*

This seems to relate to a largely unfounded perception that, once trained, the GPNs would simply ‘follow the money’. Whilst some of the GPNs were clearly ‘economically mobile’, the vast majority of the GPNs were viewed as being driven by the perceived quality of their terms and conditions rather than purely by financial concerns.

#### Green shoots of cultural change

The effective provision of GPN education and training for the neophyte GPNs clearly required a good ‘fit’ between the perceived needs of the practice and the GP partners and the developmental needs of the GPNs involved. The most interesting aspect of the study was that in spite of all their reservations, a number of the GPs interviewed did appear to be moving from a “why *should* we invest in our practice nursing workforce?” perspective to a “why *shouldn’t* we invest in our practice nursing workforce?” perspective.

From a workforce perspective, this subtle but crucial change in emphasis may be seen as vindication of the success of the ATPS and the CoP that developed as a result. Some of the more forward thinking GPs had realised the benefit to them of a well-educated and well-supported GPN workforce. As this GP noted, when asked about the ATPS:

*"…This* [ATPS] *has been a massive but necessary change when you think that general practices are still, the majority, are very much 'corner shops' all doing their own thing…"*

When asked to look into the future:

*"… I think in the future you're going to get practices working together to employ nurses to meet the demand. It will have to happen … but they may have to work across practices…"*

As a final thought, some of the respondents were already thinking about the longer term impact of the ATPS:

*"What's next then? Well, in my mind the* [long term] *plan would be to make the ATPS, now they're well-established, to make them proper educational hubs for doctors and nurses alike …"*

## Discussion

The well-documented and widely reported fall in the number of GP trainees has meant that the GPN role has assumed a much greater significance within the provision of general practice services. The realisation that a substantial number of existing GPNs are nearing retirement age at a time when more and more services are being moved into primary care has raised the prospect of a ‘perfect storm’ within the general practice workforce. There are a number of issues that affect the ability of general practice as a whole to address this impending ‘storm’. Primarily, these relate to the prevailing culture within general practice. This is complex and socially constructed, with a medical *hegemony* that is both extremely powerful and firmly embedded [25, 26]. There is a widespread agreement that the number of GPNs needs to be increased if primary care is to meet future demand; however the culture of general practice makes this difficult to achieve [27].

The RCGP *Roadmap to Excellence* report [2] clearly articulated the need to attract more new graduate nurses into general practice if the predicted increases in workload and complexity of care are to be satisfactorily addressed in the future. In order to address this predicted shortfall in GPN numbers, there has been a move to increase the numbers of new graduate nurses entering general practice. This has met with some resistance, and to better understand the situation, we need to look at the historical and cultural antecedents [1, 9–13]. Traditionally there has been little or no incentive for GPs to consider employing new graduate nurses. GPNs are employed by the partners and not the NHS, and are therefore seen as a ‘cost’ to the business [4]. However, GPNs contribute a significant amount of income to general practice through meeting UK Quality Outcomes Framework (QOF) monitoring targets for long term conditions [6, 7]. Consequently, GPs prefer to recruit experienced nurses, rather than invest in new graduate nurses and absorb the costs involved in providing them with the education and training required for the role. When a GPN post becomes vacant, there is evidence of a GPN recruitment ‘merry go round’ in which new GPNs are often appointed by being ‘poached’ from other GP practices [13].

The findings from a companion paper [27], which looked at student nurse perceptions of general practice placements, found that graduating student nurses are often under the (mis)impression that they ‘need’ to have secondary care experience before applying for a GPN post [10]. These myths have had the effect of both dissuading new graduates from applying for GPN posts, and continuing to ‘excuse’ GPs from considering them. As with most myths, they have assumed a certain degree of truth. Historically, this has resulted in a situation in which new graduate nurses do not feature in general practice. The overarching philosophy of the ATPS is therefore to promote lasting cultural change within general practice. Through the ATPS, GPs are fully supported to ‘grown their own’ GPNs by recruiting new graduate nurses. As the ATPS becomes fully embedded, the idea of ‘growing your own’ is beginning to change the prevailing culture within general practice [9, 10]. In order to better appreciate the theoretical context in which this cultural shift is currently taking place, the ATPS may be considered in terms of an embryonic CoP [22]. From a pedagogical perspective, the GPs learn about student nurses through their participation in the shared social practices of the general practice team [23]. It may be argued that the GPs’ learning is therefore seen in terms of a process in which they learn to view the students as ‘proto-GPNs’, through the students’ adoption of a new, albeit temporary identity. The CoP also provided the students with the opportunity to positively influence the social context in which the learning takes place [22]. From a cultural perspective, this social and contextual learning is clearly vital in positively influencing the GPs perceptions of ‘new’ nurses. According to Lave and Wenger, the students’ emerging identity will enable the GPs to modify their perceptions of, and attitudes towards the student nurses and their role in general practice. The regular interaction with the student nurses through the CoP will, it is argued; positively influence the GPs’ perceptions over the benefits of recruiting new graduate nurses. The successful facilitation of a Community of Practice (CoP) for placements within general practice is used to provide GPs with the knowledge and understanding needed to gain a much more useful and authentic insight into the benefits of ‘modern’ nurse education [13].

## Conclusions

There is some, albeit fragile, evidence within this study of a cultural shift in general practice towards the proactive recruitment of new graduate nurses. This has been predicated upon the development of a CoP, which has enabled the GPs to develop a better understanding of the benefits to their practice of employing new graduate nurses. This understanding has emerged through socially-situated interaction mediated by the CoP [21]. This degree of culture change has taken time, patience and a great deal of persistence. Despite the challenges, the evidence is that the ATPS scheme is beginning to ‘bear fruit’, and there is a subtle but discernible move by GPs from a ‘why *would* we?’ to ‘why *wouldn’t* we?’ recruit new graduate nurses into general practice and invest in their education and training.

## Additional file

Additional file 1: Interview schedule. (DOCX 14 kb)

## Abbreviations

ACP: Advanced Clinical Practitioner
ATPS: Advanced Training Practice Scheme
CoP: Community of practice
GP: General Practitioner
GPN: General Practice Nurse
HCA: Health care assistant
HEE: Health Education England
HEI: Higher Education Institution
NHS: National Health Service (UK)
NTHI: National Training Hubs Initiative
QNI: Queen’s Nursing Institute
QOF: Quality Outcomes Framework
RCGP: Royal College of General Practitioners
UK: United Kingdom
