# Supplementary material for: GP/GPN partner* perspectives on clinical placements for student nurses in general practice: can a community of practice help to change the prevailing culture within general practice?
Source: BMC Fam Pract. 2018 Sep 8;19:156. doi: 10.1186/s12875-018-0842-2 (PMC6128980; doi:10.1186/s12875-018-0842-2)
Supplement: Supplementary file 1 — Interview schedule. (DOCX 14 kb) [file 12875_2018_842_MOESM1_ESM.docx]

**Interview schedule: GPs and GPN partners**

1. Introductions and preamble
2. How many student nurses you had on clinical placement?
3. What did you know about modern nurse education before they started?
4. What were your thoughts before the students arrived on placement? What factors influenced your decision to take a student?
5. What are your thoughts about the student nurse placements now?
6. Have your views changed following the placements? Please can you expand?
7. Would you consider recruiting a newly graduated nurse into general practice? Please can you expand?
